# Impact of birth weight on long-term growth and development in children: evidence from a nationwide cohort study

**DOI:** 10.1186/s12887-026-06670-4

**Published:** 2026-03-04

**Authors:** Sung-Hoon Chung, Jae Woo Lim, Tae Hyeong Kim, Soon Min Lee, Jae Won Shim, Jang Hoon Lee, Jin A Lee, Yun Sil Chang, Chang-Ryul Kim

**Affiliations:** 1 Department of Pediatrics, Kyung Hee University College of Medicine, Kyung Hee University Hospital at Gangdong, Seoul, Korea; 2 Department of Pediatrics, Konyang University College of Medicine, Daejeon, Korea; 3 Department of Pediatrics, Yonsei University College of Medicine, Seoul, Korea; 4 Department of Pediatrics, Kangbuk Samsung Hospital, Sungkyunkwan University School of Medicine, Seoul, Korea; 5 Department of Pediatrics, Ajou University School of Medicine, Suwon, Korea; 6 Department of Pediatrics, Seoul National University-Seoul Metropolitan Government Boramae Medical Center, Seoul National University College of Medicine, Seoul, Korea; 7 Department of Pediatrics, Samsung Medical Center, Sungkyunkwan University School of Medicine, Seoul, Korea; 8 Department of Pediatrics, Hanyang University College of Medicine, Seoul, Korea; 9 Department of Pediatrics, Hanyang University Guri Hospital, Kyoungchun-ro 153, Guri-si, Gyeonggi-do 11923 Korea

**Keywords:** Birth weight, Growth and development, Child development, Infant, Low birth weight, Cohort study

## Abstract

**Background:**

Birth weight (BW) is a well-established key marker of early health, but its long-term influence on growth and development in the general pediatric population remains uncertain. This study aimed to assess the longitudinal association of BW on growth and neurodevelopment from infancy to six years of age using nationwide population-based data from Korea.

**Methods:**

We analyzed data from 3,033,843 children born between 2013 and 2020 who participated in the Korean National Health Insurance Service infant and child health screening program. Participants were stratified into five birth weight categories (< 1,000; 1,000–1,499; 1,500–2,499; 2,500–3,999; and ≥ 4,000 g) and followed across seven screening rounds. Growth and neurodevelopmental outcomes were assessed using standardized anthropometric measures and the Korean Developmental Screening Test for Infants and Children (K-DST), respectively. Longitudinal patterns were examined at each round, and the primary analysis focused on outcomes at 30–36 months using multivariable logistic regression to identify risks for growth failure and screening-positive developmental delay.

**Results:**

Across screening rounds, children with lower BWs, especially those under 1,500 g, showed consistently lower growth percentiles and higher rates of K-DST screen-positive results. At 30–36 months, infants with BW < 1,000 g had substantially higher odds of growth failure across all three anthropometric measures (height, weight, and head circumference) (aOR range 25.2–43.1 vs. ≥ 2,500 g; all *p* < 0.001) and of screening positive for developmental delay (K-DST < − 2 SD) (aOR 3.62, 95% CI 3.12–4.21, *p* < 0.001). Male sex and neonatal morbidities such as bronchopulmonary dysplasia, periventricular leukomalacia, and hypoxic-ischemic encephalopathy were also associated with growth failure and developmental delay at 30–36 months. Disparities in growth and development persisted across screening rounds, particularly among the < 1,500 g BW groups.

**Conclusions:**

Lower BW, particularly < 1,500 g, was associated with higher odds of growth failure and screening-positive developmental risk at 30–36 months, with disparities in growth and development persisting through early childhood.

**Supplementary Information:**

The online version contains supplementary material available at 10.1186/s12887-026-06670-4.

## Introduction

Birth weight (BW) is a key marker of early health and has been associated with both immediate neonatal outcomes and subsequent growth and developmental patterns [1, 2]. Low birth weight infants (LBWIs, BW < 2,500 g), even when delivered at term, often struggle with postnatal growth and are more likely to show developmental concerns, including delays in motor or cognitive skills, as well as conditions such as autism spectrum disorder or attention deficit hyperactivity disorder [2, 3]. Children below the 25th percentile for BW carry an elevated risk of neurological and developmental problems, and the risk rises sharply for those below the 3rd percentile [4]. Extremely low birth weight infants (ELBWIs, BW < 1,000 g) are particularly vulnerable, with frequent difficulties in cognition, movement, and attention that can require ongoing educational or therapeutic support [5].

While the link between BW and immediate neonatal outcomes is well recognized, its association with later growth and development across childhood has not been fully clarified [6, 7]. Most previous studies have centered on specific high-risk groups, such as preterm or very low birth weight infants (VLBWIs, BW < 1,500 g). Far fewer studies have examined outcomes across the full birth-weight spectrum in the general pediatric population, including children with normal BW [8–10]. Furthermore, although previous studies have provided important insights into BW–related health outcomes, few have described both long-term growth patterns and developmental trajectories across infancy and early childhood within the same nationwide population-based dataset [11, 12].

While BW is a well-recognized marker of early health, its association with long-term growth and neurodevelopment across childhood has not been fully clarified [13, 14]. Most previous studies have focused on specific high-risk groups or short follow-up periods, which limits understanding of longitudinal trajectories across the full BW spectrum [15, 16]. In addition, evidence regarding sex differences in growth and neurodevelopment remains inconsistent. Some population-based studies suggest greater vulnerability among male infants, whereas others report small or domain-specific differences, and conclusions across studies are not uniform [17, 18]. These gaps indicate the need for large-scale, longitudinal analyses with sufficient power to examine persistent disparities and sex-specific patterns over time. Using nationwide screening data from infancy to six years of age, the present study evaluated long-term growth and developmental trajectories across all BW categories, with particular attention to differences by sex.

## Methods

### Data source and study cohort

This study was designed as a population-based retrospective cohort study using data from the Korean National Health Insurance Service (NHIS) infant and child health screening program, a nationwide population surveillance system covering nearly the entire pediatric population in Korea. A birth cohort was defined at the time of delivery, including children born between January 1, 2013, and December 31, 2020, and individuals were followed longitudinally through routinely scheduled screening examinations to evaluate prespecified growth and developmental outcomes. This screening program systematically assesses child health at seven time points: 4–6 months (1st), 9–12 months (2nd), 18–24 months (3rd), 30–36 months (4th), 42–48 months (5th), 54–60 months (6th), and 66–71 months (7th). Because the screening program is age-based, follow-up at each stage included only children who had reached the corresponding age at the time of analysis; therefore, not all children born in later years contributed data to later screening rounds. Participants were categorized into five groups based on BW: <1,000 g, 1,000–1,499 g, 1,500–2,499 g, 2,500–3,999 g, and ≥ 4,000 g. Both sexes were included to allow comprehensive analysis across the full spectrum of BW. Information on BW was obtained either through diagnostic codes from the International Classification of Diseases, 10th Revision (ICD-10), entered by the hospital at birth, or from parent-completed questionnaires administered as part of the screening program. Major neonatal morbidities, including respiratory distress syndrome (RDS), bronchopulmonary dysplasia (BPD), intraventricular hemorrhage (IVH), periventricular leukomalacia (PVL), necrotizing enterocolitis (NEC), and hypoxic-ischemic encephalopathy (HIE), were identified using ICD-10 diagnostic codes entered by attending clinicians. Diagnoses were based on clinical judgment following established neonatal criteria.

### Data collection and variables

Anthropometric data (height, weight, and head circumference) were obtained at each screening. Percentiles were automatically calculated in the NHIS system, using the WHO Growth Standards for children under two years of age and the 2007 Korean National Growth Charts for older children. These reference curves are the official standards recommended by national pediatric screening guidelines in Korea, as they are population-specific and reflect growth trends in Korean children [19]. Growth failure was defined for each parameter as a value below the 3rd percentile according to these standards.

Developmental status was assessed using the Korean Developmental Screening Test for Infants and Children (K-DST) [20–22], which evaluates six developmental domains: gross motor, fine motor, cognition, language, sociality, and self-care. The self-care domain was evaluated from 18 months, and because K-DST assessments are not conducted at the 1st screening (4–6 months), developmental data were available from the 2nd screening (≥ 9 months). The K-DST categorizes developmental progress into four levels according to standard deviation (SD) scores: advanced ( > + 1 SD), peer-level (− 1 to + 1 SD), need for follow-up (− 2 to − 1 SD), and need for further testing ( < − 2 SD). In preterm infants, corrected age was applied until 24 months for both growth and developmental assessments, after which chronological age was used.

### Study design and outcome measures

The primary outcome was defined as growth failure (below the 3rd percentile for height, weight, and head circumference) and developmental risk status (screen-positive on the K-DST) at the 4th screening session (30–36 months). This period was prespecified as the primary endpoint because it is the first stage with fully standardized developmental assessments after preterm infants transition from corrected to chronological age, representing a clinically relevant window when persistent differences in growth and development become apparent [23, 24]. Furthermore, assessments at this stage provide higher predictive validity for long-term functional outcomes. Evaluations at all other screening rounds, including the final assessment at 66–71 months (approximately 6 years), were defined as secondary outcomes used to describe longitudinal growth and developmental patterns. For each screening round, analyses were limited to children with recorded BW and available anthropometric or K-DST data at that round. Children lacking BW information or outcome data at the 4th screening were excluded from the respective analyses. Accordingly, multivariable regression analyses were restricted to children with recorded BW and complete anthropometric and K-DST data at the 4th screening.

### Statistical analysis

For continuous variables, including height, weight, and head circumference percentiles, and categorical variables, such as the percentage of children below the 3rd percentile, one-way ANOVA and chi-square tests were used, respectively, to compare differences across BW groups. We then fitted multivariable logistic regression models to identify factors associated with growth failure and developmental delay at 30–36 months. For growth failure, models were run separately for height, weight, and head circumference. In all models, the ≥ 2,500 g group was the reference, as this threshold denotes normal BW. In the descriptive analyses (figures and percentages), the ≥ 4,000 g group is shown separately. In the regression models, the ≥ 4,000 g subgroup was combined with 2,500–3,999 g to enhance stability and interpretability because it comprised a small share of the cohort (3.1%) and showed profiles comparable to the 2,500–3,999 g group across screenings. Independent variables were BW category, sex, and major neonatal morbidities (RDS, BPD, IVH, PVL, NEC, HIE). Adjusted odds ratios (aORs) with 95% confidence intervals (CI) and *p*-values are reported; univariable estimates are not displayed. Statistical significance was set at *p* < 0.05. All statistical analyses were performed using SAS version 9.4 (SAS Institute, Cary, NC, USA). Gestational age (GA) was not included as a covariate because GA and BW were available only as ICD-10 based categories at birth, which did not allow derivation of BW-for-GA indicators (e.g., small for gestational age (SGA) or large for gestational age) or continuous GA modeling. We therefore used BW as the main exposure and interpreted the estimates as associations. Missing data were managed using complete-case analysis. The NHIS infant and child health screening program employs standardized data collection protocols with systematic validation. Individuals with missing BW or outcome data at any given screening were excluded from analyses for that round. This study is reported in accordance with the STROBE guidelines for observational studies.

### Ethical considerations

This study utilized data from the NHIS database (NHIS-2021-4-007), which ensures the protection of patient privacy by anonymizing all identifiable information, including claim numbers, individual identifiers, and organizational identification numbers. These variables were randomly re-generated within the NHIS database to safeguard personal data. The study protocol was reviewed and approved by the Institutional Review Board (IRB) of Hanyang University Guri Hospital (approval No. GURI 2020-08-026). Due to the retrospective nature of the study and the use of de-identified data, the requirement for informed consent was waived by the IRB.

## Results

**Fig. 1:**
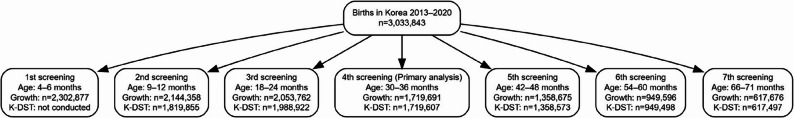
Participant flow diagram across seven infant and child health screening rounds. The diagram shows the distribution of participants across the seven standardized screening rounds conducted between 2013 and 2020. The initial cohort comprised all live births in Korea during this period (*n* = 3,033,843). At each screening stage, growth measurements and developmental assessments using the Korean Developmental Screening Test for Infants and Children (K-DST) were obtained from participants who had reached the appropriate age. Children with unknown birth weight at each screening were excluded from analyses for that specific round. The fourth screening (30–36 months of age) was designated as the primary analysis point

The study included 3,033,843 newborns in Korea from 2013 to 2020, with the number of births declining from 442,418 in 2013 to 279,093 in 2020. The distribution of BWs was as follows: 2,762,525 (91.1%) weighed 2,500–3,999 g, 151,527 (5.0%) weighed 1,500–2,499 g, 12,303 (0.4%) weighed 1,000–1,499 g, 6,741 (0.2%) weighed < 1,000 g, and 94,194 (3.1%) weighed ≥ 4,000 g (Supplementary Table 1). Participation rates in the infant and child health screening program across the seven stages were 76.0% (*n* = 2,306,370) at the 1st, 70.8% (*n* = 2,147,767) at the 2nd, 67.8% (*n* = 2,057,712) at the 3rd, 56.8% (*n* = 1,723,218) at the 4th, 44.9% (*n* = 1,361,443) at the 5th, 31.4% (*n* = 951,390) at the 6th, and 20.4% (*n* = 618,747) at the 7th screening. Participation rates varied by BW, with notably lower rates observed in the < 1,000 g and 1,000–1,499 g groups compared to the ≥ 2,500 g group across all screening rounds (17.8%, 44.9%, and 77.4%, respectively, at the 1st screening), although this disparity narrowed at the primary analysis point of the 4th screening (36.7%, 49.7%, and 57.9%, respectively) (Supplementary Table 2). Growth data were collected at all seven rounds, while developmental assessments (K-DST) were conducted from the 2nd round onward. Children with unknown BW at each screening were excluded from the analysis for that specific round. Specifically, for the primary analysis at the 4th screening (30–36 months), the final analytic sample consisted of 1,719,691 children for growth assessments and 1,719,607 children for developmental assessments, after excluding those with unknown BW. The detailed participant flow and stage-specific sample sizes are illustrated in Fig. 1.

The cohort comprised 1,556,392 male infants (51.3%). Neonatal morbidities showed a striking inverse relationship with BW. ELBWIs had the highest rates of major neonatal complications, including RDS (94.0%), BPD (54.4%), IVH (24.9%), and NEC (17.8%). Infants weighing 1,000–1,499 g also demonstrated markedly elevated rates across all morbidity types, ranging from 2.2% for hypoxic-ischemic encephalopathy to 72.3% for respiratory distress syndrome. In contrast, infants with BW ≥ 2,500 g had substantially lower rates of these complications, with prevalences below 5.7% across all morbidity types (Table 1).

**Table 1:** Baseline characteristics of the birth cohort by birth weight group

Birth weight (g)	n (%)	Male (%)	RDS (%)	BPD (%)	IVH (%)	PVL (%)	NEC (%)	HIE (%)
< 1,000	6,741 (0.2)	51.3	94	54.4	24.9	8.1	17.8	2.9
1,000–1,499	12,303 (0.4)	49	72.3	22.8	12.1	7.3	6.8	2.2
1,500–2,499	151,527 (5.1)	53.4	16.6	1	2.3	1.2	0.6	0.8
2,500–3,999	2,762,525 (93.6)	48.9	3.7	0.05	0.1	0.06	0.04	0.1
≥ 4,000	94,194 (3.2)	64.6	5.7	0.05	0.2	0.1	0.04	0.1
Unknown	6,553 (0.2)	49.7	21.3	4.7	4.6	2	1.3	1.9
Total	3,033,843 (100)	51.3	5	0.3	0.4	0.2	0.1	0.2

Birth weight data are presented as n (%); all other data are presented as percentages (%)

*RDS* Respiratory distress syndrome, *BPD* Bronchopulmonary dysplasia, *IVH* Intraventricular hemorrhage, *PVL* Periventricular leukomalacia, *NEC* Necrotizing enterocolitis, *HIE* Hypoxic ischemic encephalopathy

### Longitudinal growth patterns by BW

**Fig. 2:**
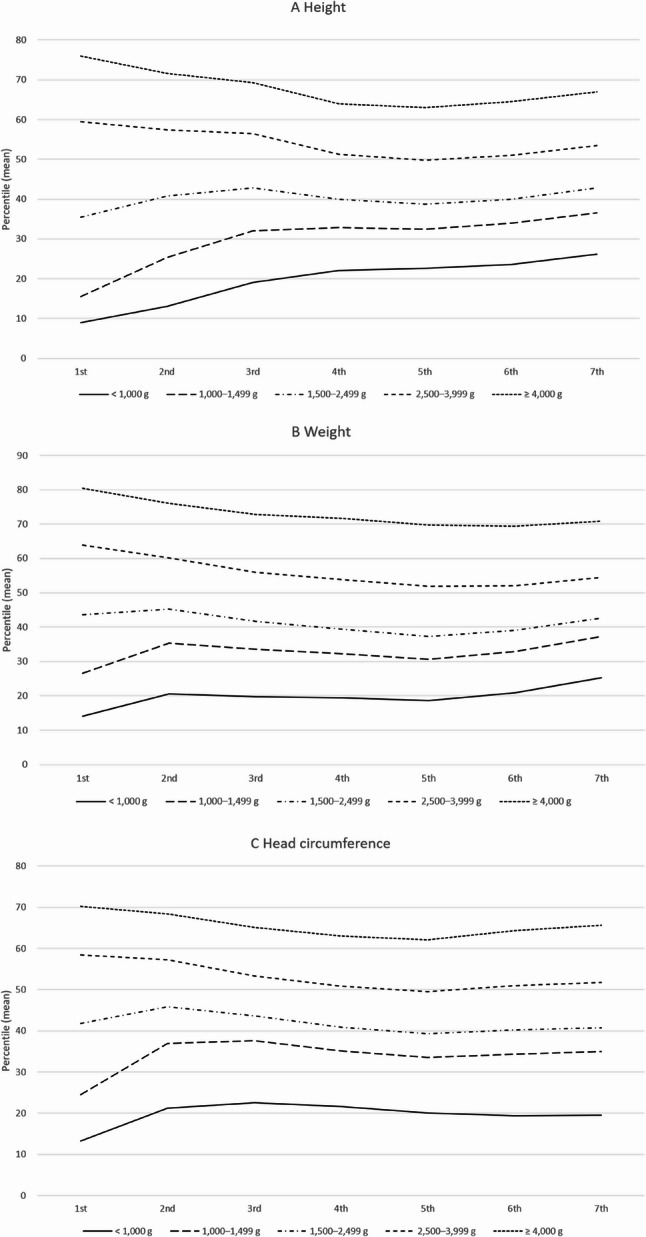
Mean percentiles of growth parameters across the seven screening rounds by birth weight group. **A** Height, **B** Weight, **C** Head circumference. Sample sizes (*n*) by screening round are shown. For example, at the 1st screening, the number of participants in each birth weight group was: <1,000 g, *n*=1,199; 1,000–1,499 g, *n*=5,530; 1,500–2,499 g, *n*=80,365; 2,500–3,999 g, *n*=2,096,454; and ≥4,000 g, *n*=119,329. Detailed counts for all screening rounds are available in Supplementary Table 8

The mean percentiles of height, weight, and head circumference from the 1st to the 7th infant and child health screenings are presented in Fig. 2, grouped by BW categories: <1,000 g, 1,000–1,499 g, 1,500–2,499 g, 2,500–3,999 g, and ≥ 4,000 g. Across all growth parameters, there were statistically significant differences among BW groups at each screening stage (all *p* < 0.001; determined by one-way ANOVA). In general, the disparities in mean percentiles between BW groups decreased through the 3rd screening but then remained relatively stable from the 4th screening onward, with each BW group following a consistent growth trajectory. Infants in the higher BW categories continued to maintain higher mean percentiles across all growth parameters, whereas those in the lower BW categories remained at lower percentiles. This pattern emphasizes the lasting influence of BW on growth, with initial differences becoming steady over time and reflecting persistent disparities in growth outcomes across groups.

**Fig. 3:**
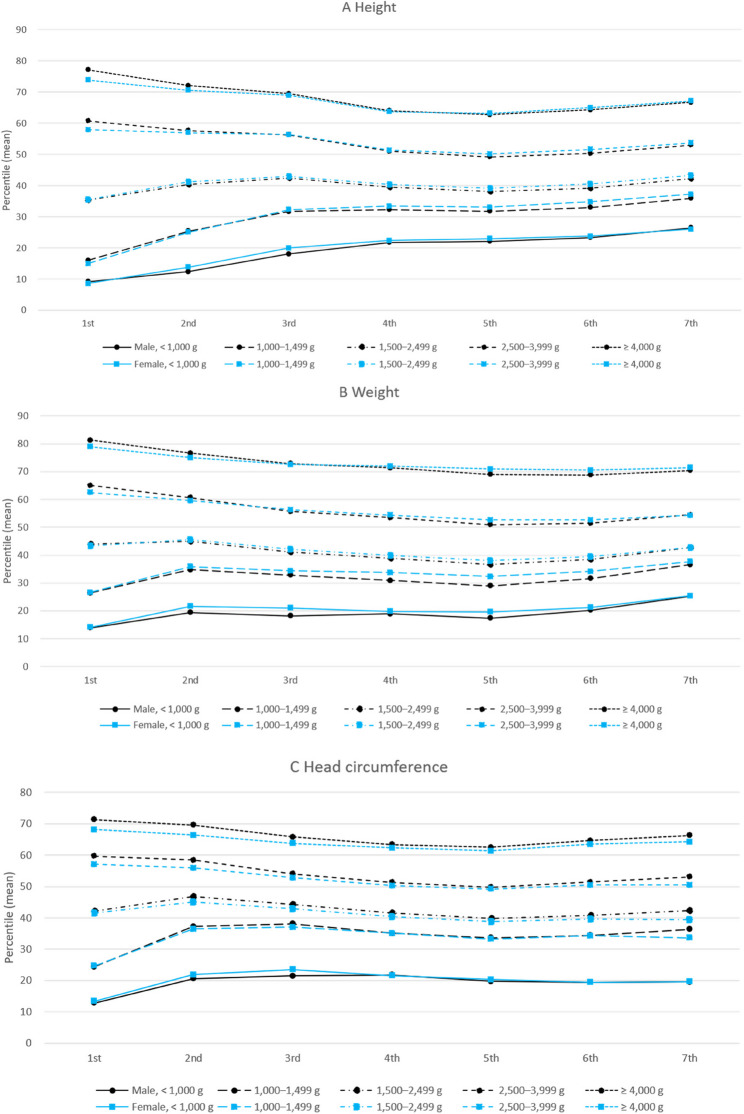
Mean percentiles of growth parameters by birth weight group and sex across the seven screening rounds. **A** Height, **B** Weight, **C** Head circumference. Sample sizes (*n*) by screening round and sex are shown. For example, at the 1st screening, the number of male participants in each birth weight group was: <1,000 g, *n*=550; 1,000–1,499 g, *n*=2,766; 1,500–2,499 g, *n*=52,052; 2,500–3,999 g, *n*=1,075,360; and ≥4,000 g, *n*=55,228. Corresponding counts for females and other screening rounds are provided in Supplementary Table 8

Figure 3 presents the same percentile distributions stratified further by gender. Similar patterns were observed for both male and female infants. Infants in higher BW categories consistently maintained higher mean percentiles for height, weight, and head circumference across all screenings, while those in lower BW categories remained at lower percentiles.

### Percentage of growth measurements below the 3rd percentile by BW

**Fig. 4:**
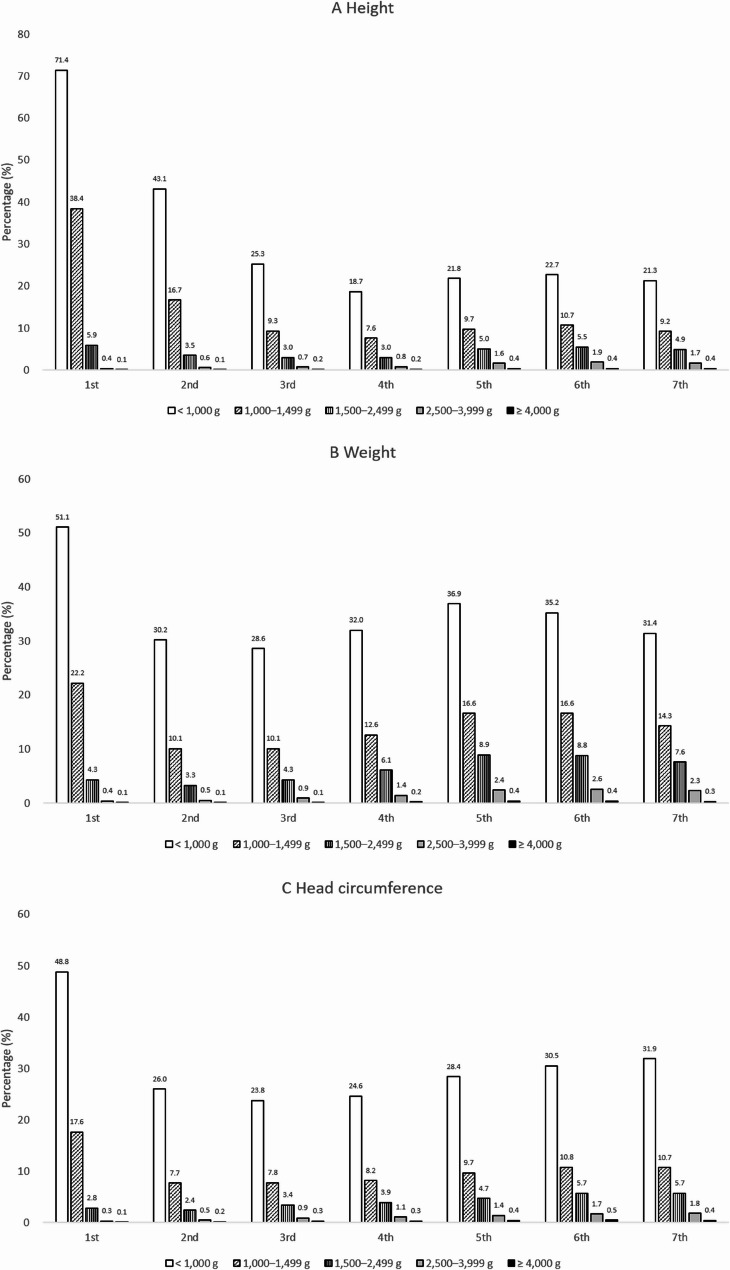
Percentage of children in each birth weight group with growth parameters below the 3rd percentile across the seven screening rounds. **A** Height, **B** Weight, **C** Head circumference. Sample sizes (*n*) by screening round are identical to those in Fig. 2. Detailed counts by birth weight group for all rounds are available in Supplementary Table 8

Figure 4 shows the percentage of infants in BW group whose height, weight, and head circumference measurements were below the 3rd percentile from the 1st to the 7th infant health screenings. Infants in the lowest BW category (< 1,000 g) consistently had the highest percentage of measurements below the 3rd percentile across all screening stages. At the 1st screening, 71.4% of infants in this group had height measurements below the 3rd percentile, while 51.1% were below the 3rd percentile for weight and 48.8% for head circumference. Over time, the percentage of infants below the 3rd percentile gradually declined across all BW groups, and the gap between groups became less pronounced. After the 4th screening, the rates tended to level off, with no major shifts observed in later screenings. In the secondary analysis of outcomes at the 7th screening (6 years), infants in the < 1,000 g group still had the highest percentage of measurements below the 3rd percentile, while those in the higher BW groups (≥ 2,500 g) showed relatively stable and low percentages, indicating that their growth remained more consistent over time.

### K-DST screen-positive (risk of developmental delay) by BW category

**Fig. 5:**
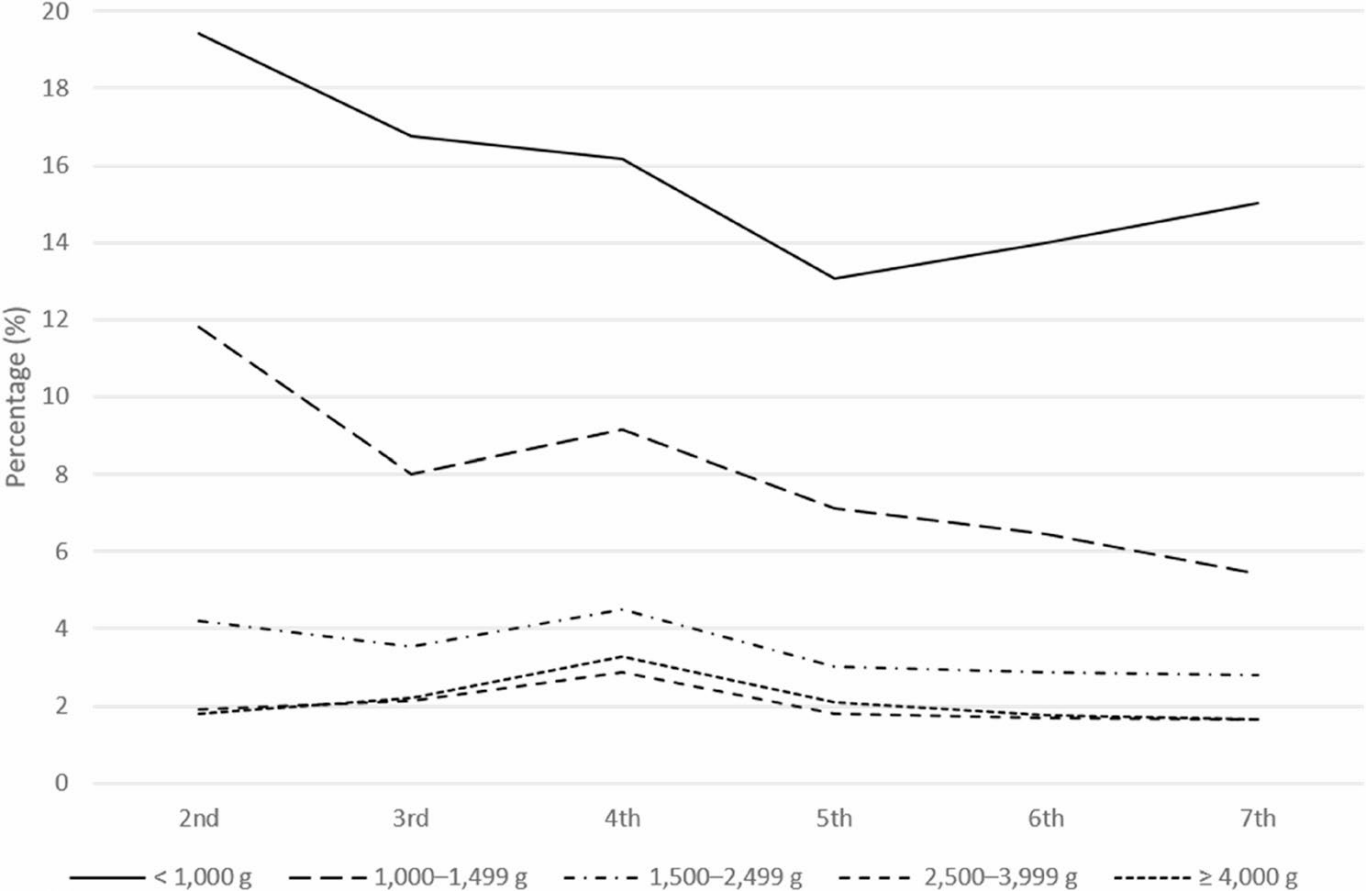
Percentage of children in each birth weight group who screened positive for developmental delay (K-DST total score <−2 SD) across the 2nd to 7th screening rounds. Analyses included participants with available K-DST results only. For example, at the 2nd screening, the number of participants in each birth weight group was: <1,000 g (*n*=1,528); 1,000–1,499 g (*n*=5,492); 1,500–2,499 g (*n*=97,869); 2,500–3,999 g (*n*=1,651,806); and ≥4,000 g (*n*=63,160). Detailed counts for all screening rounds are provided in Supplementary Table 9

Developmental delays assessed by the K-DST were most prevalent among infants with lower BWs, particularly those in the < 1,000 g category. At the 2nd screening, 19.4% of infants in this group required further evaluation, and although the percentage gradually declined over time, it remained at 15.0% by the 7th screening as a secondary outcome. A similar trend was observed in the 1,000–1,499 g group, where the percentage of developmental delays decreased from 11.8% to 5.4%. In contrast, infants with BWs ≥ 2,500 g consistently showed lower rates of developmental delays, remaining below 3% throughout the screenings, while the 1,500–2,499 g group showed slightly higher percentages, peaking at 4.5% at the 4th screening (Fig. 5).

**Fig. 6:**
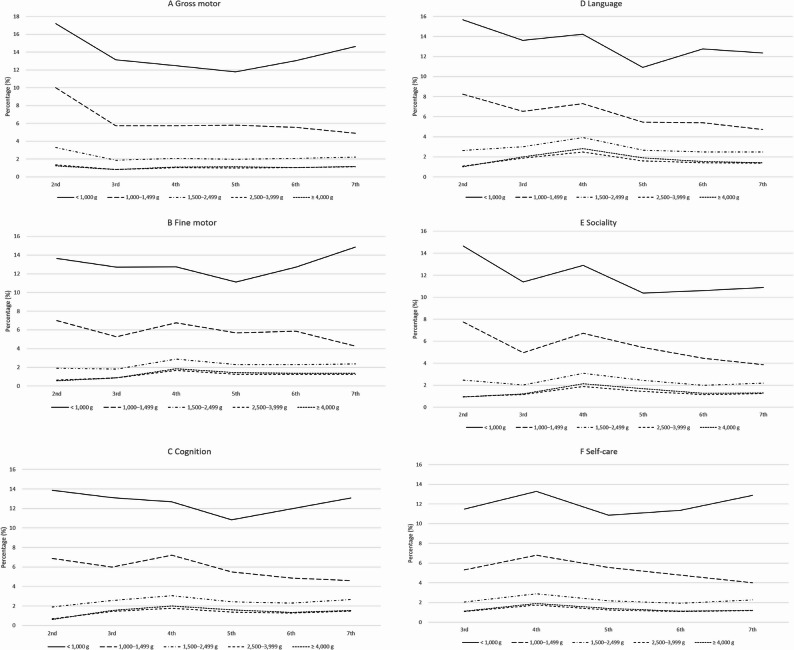
Percentage of children in each birth weight group who screened positive for developmental delay in individual K-DST domains. **A** Gross motor, **B** Fine motor, **C** Cognition, **D** Language, **E** Sociality, **F** Self-care (defined as domain scores <−2 SD). Analyses included participants with available K-DST results only

When examining developmental domains separately, delays in gross motor, fine motor, cognition, language, social skills, and self-care followed a similar pattern. Infants in the < 1,000 g category had the highest delay rates across all domains, with gross motor and language skills showing particularly high percentages at the 2nd screening. Although delay rates tended to decline over time, a reversal of this trend was observed in the < 1,000 g group, where the percentages began to increase again from the 5th to the 7th screening. In contrast, infants in the 1,000–1,499 g group exhibited a steady decline in delay rates across screenings, with values by the 7th screening approximately half of those at the 2nd. Meanwhile, infants in the ≥ 1,500 g groups showed minimal variation, with delay rates remaining mostly below 3% across all domains (Fig. 6). Detailed numerical data corresponding to each figure, including outcomes in the ≥ 4,000 g group, are provided in Supplementary Tables 3–7.

### Primary analysis of factors associated with growth failure and developmental delays at 30–36 months

At 30–36 months, primary multivariable models demonstrated graded associations between lower BW and growth failure across height, weight, and head circumference, with consistent findings for K-DST based developmental delay (Tables 2 and 3). Using infants with BW ≥ 2,500 g as the reference, those with BW < 1,000 g had markedly higher odds of growth failure (aOR 25.16 for height, 43.10 for weight, and 30.20 for head circumference; all *p* < 0.001) as well as screening positive for developmental delay (aOR 3.62, 95% CI 3.12–4.21). The 1,000–1,499 g and 1,500–2,499 g groups also showed stepwise increases in risk across all outcomes. Male sex was associated with higher odds of growth failure (height and weight) and screening positive for developmental delay, but with lower odds of head circumference <3rd percentile. Among neonatal morbidities, BPD, NEC, and HIE were consistently associated with adverse outcomes across anthropometric and developmental measures, whereas PVL showed strong associations primarily with screening-based developmental delay risk. RDS was not significantly associated with growth failure but remained positively associated with screening positive for developmental delay.

**Table 2:** Multivariable regression analyses for growth failure (< 3rd percentile) at 30–36 months across three anthropometric measures: adjusted odds ratios (95% CI) compared with univariate estimates for birth weight

Outcome	OR (95% CI)					
	Height	*p*-value	Weight	*p*-value	Head circumference	*p*- value
Birth Weight						
< 1,000 g						
Univariate	27.90 (25.18–30.91)	< 0.001	34.12 (31.84–36.57)	< 0.001	30.06 (27.89–32.41)	< 0.001
Adjusted	25.16 (21.49–29.46)	< 0.001	43.10 (37.60–49.50)	< 0.001	30.20 (25.80–35.30)	< 0.001
1,000–1,499 g						
Univariate	10.00 (9.09–11.01)	< 0.001	10.45 (9.73–11.23)	< 0.001	8.23 (7.55–8.98)	< 0.001
Adjusted	9.60 (8.53–10.81)	< 0.001	12.10 (11.00–13.40)	< 0.001	8.15 (7.25–9.18)	< 0.001
1,500–2,499 g						
Univariate	3.80 (3.64–3.97)	< 0.001	4.71 (4.57–4.86)	< 0.001	3.74 (3.60–3.88)	< 0.001
Adjusted	3.84 (3.68–4.01)	< 0.001	4.70 (4.54–4.86)	< 0.001	3.60 (3.45–3.76)	< 0.001
≥ 2,500 g	1.00		1.00		1.00	
Sex (Male)	1.21 (1.18–1.25)	< 0.001	1.20 (1.05–1.36)	< 0.001	0.90 (0.87–0.92)	< 0.001
RDS	0.90 (0.80–1.01)	0.065	0.92 (0.82–1.03)	< 0.001	0.92 (0.81–1.04)	< 0.001
BPD	1.17 (1.01–1.35)	0.036	1.20 (1.05–1.36)	0.006	1.18 (1.02–1.37)	0.020
IVH	1.05 (0.90–1.23)	0.513	1.00 (0.87–1.16)	0.582	1.10 (0.95–1.28)	0.092
PVL	1.30 (1.05–1.60)	0.014	0.95 (0.78–1.16)	0.203	0.96 (0.77–1.20)	0.610
NEC	1.57 (1.30–1.89)	< 0.001	1.26 (1.06–1.50)	0.008	1.47 (1.22–1.77)	< 0.001
HIE	1.91 (1.42–2.57)	< 0.001	1.95 (1.50–2.54)	< 0.001	2.50 (1.93–3.25)	< 0.001

Values are presented as odds ratios (95% confidence interval). Univariate and adjusted odds ratios are shown for birth weight; adjusted odds ratios are shown for sex and neonatal morbidities. Adjusted odds ratios were calculated using a multivariable model including all variables listed in the table

*CI* Confidence interval, *RDS* Respiratory distress syndrome, *BPD* Bronchopulmonary dysplasia, *IVH* Intraventricular hemorrhage, *PVL* Periventricular leukomalacia, *NEC* Necrotizing enterocolitis, *HIE* Hypoxic ischemic encephalopathy

**Table 3:** Multivariable regression analysis for developmental delay (K-DST total score < − 2 SD) requiring further evaluation at the fourth infant and child health screening program (30–36 months)

Outcome	OR (95% CI)	*p*-value
Birth Weight		
< 1,000 g		
Univariate	6.48 (5.82–7.21)	< 0.001
Adjusted	3.62 (3.12–4.21)	< 0.001
1,000–1,499 g		
Univariate	3.38 (3.10–3.69)	< 0.001
Adjusted	2.34 (2.11–2.61)	< 0.001
1,500–2,499 g		
Univariate	1.58 (1.52–1.63)	< 0.001
Adjusted	1.59 (1.54–1.65)	< 0.001
≥ 2,500 g	1.00	
Sex (Male)	2.74 (2.69–2.80)	< 0.001
RDS	1.38 (1.27–1.50)	< 0.001
BPD	1.37 (1.21–1.56)	< 0.001
IVH	1.21 (1.07–1.36)	0.002
PVL	1.99 (1.71–2.31)	< 0.001
NEC	1.37 (1.15–1.62)	< 0.001
HIE	2.29 (1.88–2.79)	< 0.001

Values are presented as odds ratios (95% confidence interval). Univariate and adjusted odds ratios are shown for birth weight; adjusted odds ratios are shown for sex and neonatal morbidities. Adjusted odds ratios were calculated using a multivariable model including all variables listed in the table

*CI* Confidence interval, *K-DST* Korean Developmental Screening Test for Infants and Children, *RDS* Respiratory distress syndrome, *BPD* Bronchopulmonary dysplasia, *IVH* Intraventricular hemorrhage, *PVL* Periventricular leukomalacia, *NEC* Necrotizing enterocolitis, *HIE* Hypoxic ischemic encephalopathy

## Discussion

In this large nationwide cohort of over 3 million children, BW showed strong associations with both growth and neurodevelopment throughout early childhood. Children with lower BW, particularly those < 1,500 g, consistently showed poorer growth and higher risks of screen-positive for developmental delay on the K-DST, a trend that persisted through the secondary endpoint at six years of age. Among them, infants born with ELBW (< 1,000 g) showed the most marked and persistent deficits. These patterns are consistent with lasting differences in somatic growth and developmental outcomes linked to early physical status. In addition to BW, male sex and several perinatal complications, specifically BPD, PVL, NEC, and HIE, were associated with higher odds of growth failure and screen-positive developmental delay; these patterns are consistent with biological vulnerability and early neonatal morbidity being linked to differences in child health.

Persistent differences in growth and neurodevelopment among LBWIs are likely shaped by a range of biological and environmental influences. For instance, changes in growth hormone or IGF-1 pathways after birth, as well as metabolic challenges that arise in utero, may have an impact on later development [25–27]. Some studies suggest that early exposure to stress can alter organ function and growth patterns over time [28]. Epigenetic changes during infancy may also affect gene expression in ways that persist throughout childhood [29]. On the environmental side, conditions such as poor maternal nutrition, exposure to air pollution or noise, and living in areas with less green space during pregnancy have all been linked to reduced BW and slower postnatal growth [30]. Socioeconomic disadvantage and limited access to health services can further increase children’s vulnerability to developmental challenges [31]. Altogether, it seems that these biological and environmental factors, especially when combined, may help explain why disparities persist in this population.

The observed growth patterns demonstrated that initial differences between BW groups gradually narrowed during infancy but stabilized significantly after the 4th screening (30–36 months) for height, and after the 3rd screening (18–24 months) for both weight and head circumference. This trend suggests a critical window for catch-up growth, primarily within the first two to three years of life [32, 33]. This period is particularly important as infants born with lower BWs attempt to align their growth trajectories with those of their peers. Catch-up growth is a period of accelerated postnatal growth that occurs as infants attempt to reach age- and sex-specific normative growth curves after being born with LBW or after being born SGA. While some infants, particularly those born moderate to late preterm, appear to achieve near-normal growth, infants born at lower GAs tend to remain below standard percentiles throughout early childhood [34, 35]. Several factors influence the extent and success of catch-up growth, including the severity of BW deficit, nutritional adequacy, and early perinatal conditions. In addition, biological and genetic predispositions such as parental height, intrauterine environment, and early morbidities like BPD, NEC, or PVL may contribute to individual variation in growth trajectories [36]. Our study demonstrated that while catch-up growth occurred during early infancy, growth disparities persisted beyond the first few years of life, particularly among infants with lower GA or significant perinatal complications. Given these findings, maintaining long-term growth surveillance and nutritional intervention beyond infancy appears necessary to address these ongoing disparities [37, 38].

Previous studies have shown that lower BW is consistently associated with higher risks of developmental delays across multiple domains, including motor, cognitive, and behavioral functions [5, 39]. Our findings were consistent with previous reports, showing that infants with lower BW had higher rates of growth failure and higher odds of being identified as at developmental risk on the K-DST across all screening rounds, including the secondary analysis at 6 years. BW reflects prenatal biological status and serves as an early indicator of developmental risk [40]. However, postnatal growth trajectories also carry important prognostic information. Patterns of weight gain, linear growth, and head growth after birth have been independently associated with later cognitive, behavioral, and metabolic outcomes [41]. For example, post-discharge growth failure in VLBWIs was associated with lower developmental scores at 24 months corrected age, even after adjustment for perinatal complications [9], and inadequate head growth during and after the neonatal period predicted poorer cognitive and motor outcomes between 16 and 36 months [42]. Together, these findings suggest that while BW establishes a baseline for developmental risk, the subsequent growth trajectory is associated with how these risks manifest over time. Our longitudinal data show that after an initial period of rapid change, both growth percentiles and developmental risk scores tend to stabilize, particularly after 30 to 48 months. Although the disparity between BW groups persists throughout the screening period as shown in our secondary analyses, this stabilization underscores the clinical significance of the early years as a critical window for monitoring and supporting long-term developmental trajectories. This interpretation is supported by longitudinal evidence showing that postnatal growth velocity partially mediates the relationship between BW and neurodevelopmental outcomes, with these effects being more pronounced in infants with lower BWs [43]. However, as this study did not perform a direct individual-level correlation between growth velocity and developmental outcomes within each BW stratum, these patterns should be interpreted as population-level trends rather than a definitive causal link.

In addition to BW, male sex was associated with higher odds of growth failure in height and weight as well as developmental delay compared to females. Recent data from resource-limited settings suggest that male infants tend to follow poorer early growth trajectories, including lower height- and weight-for-age z-scores, and show limited catch-up growth by 18 months [44]. Interestingly, our findings revealed a notable discrepancy where male infants exhibited a significantly lower risk of head circumference growth failure (aOR 0.90) despite having a markedly higher risk of developmental delay (aOR 2.74). This observation suggests that preserved macrostructural brain growth, as reflected by head circumference, does not invariably equate to functional neurodevelopmental integrity in males [45]. This disparity aligns with existing evidence that male neonates are more susceptible to adverse perinatal conditions due to delayed cortical maturation and heightened inflammatory responses, including increased microglial activation during hypoxic stress, which may contribute to higher rates of cognitive impairments and learning difficulties [46]. Furthermore, neurodevelopmental performance is determined more by the qualitative integrity of white matter pathways than by quantitative volume, as functional impairments can occur without proportionate volume loss in males [47]. These qualitative vulnerabilities are further driven by sex-specific neuroinflammatory patterns that impair neural circuit maturation during critical periods [48]. Consequently, these patterns suggest that relying on head circumference alone may risk false reassurance in male infants, necessitating qualitative developmental surveillance alongside routine anthropometric monitoring.

Beyond BW and sex, perinatal morbidities, particularly PVL and HIE, show strong associations with long-term developmental delays due to their impact on white matter integrity and neural network connectivity. These conditions are associated with a range of neurodevelopmental impairments, including cognitive, motor, and behavioral disorders. The profound effects of PVL and HIE on brain development are primarily due to their disruption of white matter, which is crucial for efficient neural communication [49, 50]. This disruption can lead to impaired growth and developmental delays in affected children. Similarly, BPD and NEC are associated with systemic inflammation, metabolic stress, and prolonged intensive care, which collectively impose a substantial burden on the developing infant [51, 52]. The chronic inflammation and stress from these conditions can lead to impaired growth and neurodevelopmental outcomes, as evidenced by various studies [53, 54]. Although IVH was associated with developmental delay, it was not significantly linked to growth failure in our cohort, suggesting that its impact on somatic development may be more limited or dependent on severity.

Extending these findings, it is crucial to recognize that overall postnatal growth patterns may reflect underlying developmental vulnerabilities. While our study used height as the primary marker for somatic growth due to its reliability in long-term assessments, deficits in weight and head circumference were also evident among lower BW groups and may offer additional insight into the biological and environmental stressors affecting development. Emerging research has linked poor postnatal growth, particularly when persistent across multiple parameters, to delayed cognitive outcomes, lower school readiness, and behavioral challenges during early childhood [55]. These associations underscore that somatic growth reflects not only nutritional intake but also the overall physical condition of the child, including how well they adapt to early medical challenges. Given these findings, closely tracking various growth indicators over time could be useful for spotting children who may be more vulnerable to developmental difficulties, especially those with serious complications around the time of birth.

This study uses a large, nationally representative dataset from the Korean NHIS, allowing for the analysis of over 3 million children across a wide spectrum of BWs. The dataset spans an eight-year period (2013–2020), providing rare long-term insight into population-level trends. Repeated and standardized assessments of growth and development at multiple time points improve the reliability and consistency of the results. By examining growth and development outcomes together across the full BW range, this study offers a comprehensive perspective on early childhood health. To our knowledge, few prior studies in Korea or abroad have used such extensive, systematically collected national data to evaluate both outcomes in a single cohort. Moreover, the use of population-level data from a nationwide screening program, combined with validated national assessment tools, enhances the relevance and applicability of the findings to public health practice and policy. However, several limitations should be acknowledged. First, because the NHIS infant and child health screening program is a nationwide surveillance system rather than a cohort originally designed for research, the study was conducted retrospectively using available records. Consequently, exact counts of excluded individuals at each screening stage could not be reconstructed. Furthermore, participation rates declined significantly across screenings, from 76.0% at the 1st round to 20.4% at the 7th. This trend may introduce attrition bias; depending on whether higher-risk or lower-risk children were more likely to return, adverse outcomes could be over- or underestimated. However, participation trends varied by birth weight (Supplementary Table 2). Unlike the ≥ 2,500 g group, where participation declined over time, the < 1,500 g group showed a notable increase from the 1st to the 4th screening (e.g., 17.8% to 36.7% in the < 1,000 g group). This initially low participation is likely due to the difference between chronological and corrected age. Because the 1st screening is scheduled at 4–6 months of chronological age, caregivers of VLBWIs may skip these assessments, considering them too early for their child’s developmental stage. Consequently, the higher participation at the 30–36-month primary endpoint suggests that our analysis captures a representative sample of high-risk survivors, thereby reducing concerns about selection bias for this vulnerable group. Second, certain potential confounders, such as detailed socioeconomic factors, parental anthropometry, and home environment variables, were not available in the NHIS database. Third, regarding assessment tools, the K-DST is primarily a screening measure and may not capture the full range of neurodevelopmental outcomes with the same depth as formal diagnostic evaluations. Similarly, defining growth failure using a <3rd percentile cutoff may oversimplify growth dynamics, as continuous measures such as z-scores, growth velocity, or catch-up patterns were not analyzed. Furthermore, the prevalence of certain neonatal morbidities identified via ICD-10 codes may show discrepancies when compared with clinical registries such as the Korean Neonatal Network. While the incidence of BPD and NEC in our ELBW group is generally consistent with recent national trends, differences may arise because NHIS data rely on administrative diagnostic codes rather than standardized clinical criteria [56, 57]. Additionally, the ≥ 4,000 g group was combined with the 2,500–3,999 g group in the regression to stabilize estimates, which precluded an examination of macrosomia-specific adjusted effects. Another limitation is that primary outcomes were assessed at 30–36 months. Because growth percentiles tend to stabilize after approximately 48 months due to canalization, and some early developmental delays may resolve over time, risks identified at this stage could overestimate long-term deficits. Nevertheless, this age window remains clinically vital for early intervention, which was a primary aim of this study. Finally, we could not adjust for GA, which limits our ability to separate the effects of prematurity from fetal growth restriction. In Korean reference data, sex-specific BW percentiles vary by GA, and LBW occurs in both preterm infants and term infants who are SGA [58, 59]. Part of the observed associations may therefore reflect differences in GA distribution. Accordingly, given the retrospective nature of the study, our findings should be interpreted as associations rather than direct cause-and-effect relationships.

In conclusion, this nationwide cohort study reinforces that BW is associated with both somatic growth and developmental outcomes during early childhood. Infants with lower BWs, especially VLBWIs, remained at increased risk for growth failure and neurodevelopmental delays well beyond infancy, with disparities persisting through the final secondary assessment at 6 years. These outcomes were also associated with sex and perinatal morbidities, highlighting the complex interplay of biological and early clinical factors in early childhood health. By tracking a large, representative pediatric population through routine health screenings, this study emphasizes the importance of early monitoring and the need for individualized follow-up strategies for children with higher medical vulnerability. Given the heightened risk profile of VLBWIs, enhanced and multidisciplinary follow-up programs may be warranted to better identify ongoing problems and to provide timely interventions. These targeted approaches include regular developmental surveillance, comprehensive parental education, and improved transitions to community care, which could help mitigate long-term risks and promote healthier outcomes in this vulnerable group.

## Supplementary Information

Supplementary Material 1.

## Data Availability

The datasets analyzed during the current study are not publicly available due to National Health Insurance Service (NHIS) data use restrictions but are available from the corresponding author on reasonable request.

## Abbreviations

BW: Birth weight
LBW: Low birth weight
VLBW: Very low birth weigh
ELBW: Extremely low birth weight
SGA: Small for gestational age
NHIS: National Health Insurance Service
K-DST: Korean Developmental Screening Test for Infants and Children
RDS: Respiratory distress syndrome
BPD: Bronchopulmonary dysplasia
IVH: Intraventricular hemorrhage
PVL: Periventricular leukomalacia
NEC: Necrotizing enterocolitis
HIE: Hypoxic ischemic encephalopathy
OR: Odds ratio
CI: Confidence interval
GA: Gestational age
SD: Standard deviation
