# Supplementary material for: Impact of birth weight on long-term growth and development in children: evidence from a nationwide cohort study
Source: BMC Pediatr. 2026 Mar 4;26:292. doi: 10.1186/s12887-026-06670-4 (PMC13067422; doi:10.1186/s12887-026-06670-4)
Supplement: Supplementary file 1 — Supplementary Material 1. [file 12887_2026_6670_MOESM1_ESM.docx]

| Supplementary Table 1. Annual number of newborns categorized by birth weight in Korea (2013–2020) | | | | | | | |  |  |
| --- | --- | --- | --- | --- | --- | --- | --- | --- | --- |
| Birth weight (g) | Subjects | | | | | | | | Total |
|  | 2013 | 2014 | 2015 | 2016 | 2017 | 2018 | 2019 | 2020 |  |
| <1,000 | 912 | 890 | 912 | 862 | 856 | 814 | 796 | 699 | 6,741 |
| 1,000–1,499 | 1,626 | 1,584 | 1,751 | 1,682 | 1,535 | 1,555 | 1,403 | 1,167 | 12,303 |
| 1,500–2,499 | 19,848 | 20,937 | 21,574 | 20,811 | 19,392 | 18,232 | 18,065 | 12,668 | 151,527 |
| 2,500–3,999 | 405,653 | 404,202 | 406,269 | 376,679 | 329,709 | 303,319 | 278,955 | 257,739 | 2,762,525 |
| ≥4,000 | 13,623 | 13,765 | 14,637 | 14,000 | 11,923 | 10,681 | 9,527 | 6,038 | 94,194 |
| Unknown | 756 | 963 | 1,017 | 1,049 | 883 | 572 | 531 | 782 | 6,553 |
| Total | 442,418 | 442,341 | 446,160 | 415,083 | 364,298 | 335,173 | 309,277 | 279,093 | 3,033,843 |

| Supplementary Table 2. Participation rates in infant and child health screening programs by birth weight group | | | | | |
| --- | --- | --- | --- | --- | --- |
| Screening round | Total cohort | <1,000 g | 1,000–1,499 g | 1,500–2,499 g | ≥2,500 g |
| Births (Denominator) | 3,033,843 | 6,741 | 12,303 | 151,527 | 2,863,272 |
| 1st (4–6 mo) |  |  |  |  |  |
| Participants (n) | 2,302,877 | 1,199 | 5,530 | 80,365 | 2,211,783 |
| Rate (%) | 75.9 | 17.8 | 44.9 | 53.0 | 77.2 |
| 2nd (9–12 mo) |  |  |  |  |  |
| Participants (n) | 2,144,358 | 1,733 | 6,247 | 74,749 | 2,061,629 |
| Rate (%) | 70.7 | 25.7 | 50.8 | 49.3 | 72.0 |
| 3rd (18–24 mo) |  |  |  |  |  |
| Participants (n) | 2,053,762 | 2,346 | 6,934 | 68,727 | 1,975,755 |
| Rate (%) | 67.7 | 34.8 | 56.4 | 45.4 | 69.0 |
| 4th (30–36 mo) |  |  |  |  |  |
| Participants (n) | 1,719,691 | 2,477 | 6,116 | 57,009 | 1,654,089 |
| Rate (%) | 56.7 | 36.7 | 49.7 | 37.6 | 57.8 |
| 5th (42–48 mo) |  |  |  |  |  |
| Participants (n) | 1,358,675 | 2,033 | 4,988 | 44,826 | 1,306,828 |
| Rate (%) | 44.8 | 30.2 | 40.5 | 29.6 | 45.6 |
| 6th (54–60 mo) |  |  |  |  |  |
| Participants (n) | 949,596 | 1,473 | 3,454 | 30,944 | 913,725 |
| Rate (%) | 31.3 | 21.9 | 28.1 | 20.4 | 31.9 |
| 7th (66–71 mo) |  |  |  |  |  |
| Participants (n) | 617,676 | 952 | 2,144 | 19,700 | 594,880 |
| Rate (%) | 20.4 | 14.1 | 17.4 | 13.0 | 20.8 |
| The ≥2,500 g group includes infants weighing 2,500–3,999 g and ≥4,000 g combined, due to discrepancies in macrosomia coding in birth registry data vs. screening measurements. | | | | | |

| Supplementary Table 3. Mean percentiles of growth parameters by birth weight group across the seven rounds of the infant and child health screening program | | | | | |
| --- | --- | --- | --- | --- | --- |
| Height (mean percentile) | |  |  |  |  |
| Screening round | Birth weight (gram) | | | | |
|  | <1,000 | 1,000–1,499 | 1,500–2,499 | 2,500–3,999 | ≥4,000 |
| 1st | 8.9 | 15.5 | 35.5 | 59.4 | 76.0 |
| 2nd | 13.1 | 25.3 | 40.7 | 57.4 | 71.6 |
| 3rd | 19.1 | 32.0 | 42.8 | 56.4 | 69.3 |
| 4th | 22.1 | 32.8 | 39.9 | 51.2 | 63.9 |
| 5th | 22.6 | 32.4 | 38.7 | 49.7 | 63.0 |
| 6th | 23.5 | 33.9 | 39.9 | 51.0 | 64.5 |
| 7th | 26.2 | 36.5 | 42.8 | 53.4 | 66.9 |
|  |  |  |  |  |  |
| Weight (mean percentile) | |  |  |  |  |
| Screening round | Birth weight (gram) | | | | |
|  | <1,000 | 1,000–1,499 | 1,500–2,499 | 2,500–3,999 | ≥4,000 |
| 1st | 14.0 | 26.5 | 43.6 | 63.8 | 80.5 |
| 2nd | 20.6 | 35.3 | 45.3 | 60.2 | 76.1 |
| 3rd | 19.7 | 33.6 | 41.7 | 56.0 | 72.8 |
| 4th | 19.4 | 32.3 | 39.4 | 53.9 | 71.6 |
| 5th | 18.6 | 30.6 | 37.3 | 51.8 | 69.7 |
| 6th | 20.8 | 32.9 | 39.0 | 52.1 | 69.4 |
| 7th | 25.3 | 37.2 | 42.7 | 54.4 | 70.8 |
|  |  |  |  |  |  |
| Head circumference (mean percentile) | | |  |  |  |
| Screening round | Birth weight (gram) | | | | |
|  | <1,000 | 1,000–1,499 | 1,500–2,499 | 2,500–3,999 | ≥4,000 |
| 1st | 13.2 | 24.5 | 41.8 | 58.4 | 70.2 |
| 2nd | 21.3 | 36.9 | 45.9 | 57.2 | 68.4 |
| 3rd | 22.5 | 37.6 | 43.6 | 53.4 | 65.1 |
| 4th | 21.7 | 35.1 | 40.9 | 50.8 | 63.0 |
| 5th | 20.0 | 33.5 | 39.3 | 49.5 | 62.1 |
| 6th | 19.4 | 34.3 | 40.2 | 51.0 | 64.3 |
| 7th | 19.6 | 35.0 | 40.7 | 51.8 | 65.6 |

| Supplementary Table 4. Mean percentiles of growth parameters by birth weight group and sex across the seven rounds of the infant and child health screening program | | | | | | |
| --- | --- | --- | --- | --- | --- | --- |
| Height (mean percentile) | |  |  |  |  |  |
| Screening round | Sex | Birth weight (gram) | | | | |
|  |  | <1,000 | 1,000–1,499 | 1,500–2,499 | 2,500–3,999 | ≥4,000 |
| 1st | Male | 9.2 | 16.0 | 35.4 | 60.8 | 77.2 |
|  | Female | 8.6 | 15.0 | 35.5 | 57.9 | 73.9 |
| 2nd | Male | 12.4 | 25.4 | 40.3 | 57.7 | 72.1 |
|  | Female | 13.8 | 25.1 | 41.2 | 57.0 | 70.6 |
| 3rd | Male | 18.1 | 31.7 | 42.5 | 56.3 | 69.5 |
|  | Female | 20.0 | 32.3 | 43.0 | 56.4 | 69.0 |
| 4th | Male | 21.8 | 32.3 | 39.5 | 51.0 | 64.1 |
|  | Female | 22.4 | 33.4 | 40.3 | 51.4 | 63.7 |
| 5th | Male | 22.1 | 31.8 | 38.2 | 49.2 | 62.8 |
|  | Female | 23.0 | 33.1 | 39.1 | 50.2 | 63.2 |
| 6th | Male | 23.3 | 33.0 | 39.2 | 50.3 | 64.3 |
|  | Female | 23.8 | 34.8 | 40.6 | 51.6 | 65.0 |
| 7th | Male | 26.4 | 35.9 | 42.3 | 53.0 | 66.7 |
|  | Female | 26.0 | 37.2 | 43.3 | 53.7 | 67.2 |
|  |  |  |  |  |  |  |
| Weight (mean percentile) | |  |  |  |  |  |
| Screening round | Sex | Birth weight (gram) | | | | |
|  |  | <1,000 | 1,000–1,499 | 1,500–2,499 | 2,500–3,999 | ≥4,000 |
| 1st | Male | 13.9 | 26.4 | 43.9 | 65.1 | 81.3 |
|  | Female | 14.1 | 26.6 | 43.3 | 62.5 | 79.0 |
| 2nd | Male | 19.4 | 34.8 | 45.0 | 60.7 | 76.6 |
|  | Female | 21.7 | 35.9 | 45.6 | 59.6 | 75.0 |
| 3rd | Male | 18.2 | 32.8 | 41.1 | 55.7 | 72.9 |
|  | Female | 21.0 | 34.4 | 42.2 | 56.3 | 72.6 |
| 4th | Male | 18.9 | 30.9 | 38.8 | 53.5 | 71.4 |
|  | Female | 19.8 | 33.7 | 39.9 | 54.4 | 72.0 |
| 5th | Male | 17.4 | 29.0 | 36.6 | 50.9 | 69.0 |
|  | Female | 19.6 | 32.3 | 38.0 | 52.7 | 70.9 |
| 6th | Male | 20.2 | 31.6 | 38.4 | 51.4 | 68.8 |
|  | Female | 21.3 | 34.2 | 39.5 | 52.7 | 70.5 |
| 7th | Male | 25.3 | 36.6 | 42.8 | 54.5 | 70.4 |
|  | Female | 25.4 | 37.7 | 42.7 | 54.3 | 71.5 |
|  |  |  |  |  |  |  |
| Head circumference (mean percentile) | | | |  |  |  |
| Screening round | Sex | Birth weight (gram) | | | | |
|  |  | <1,000 | 1,000–1,499 | 1,500–2,499 | 2,500–3,999 | ≥4,000 |
| 1st | Male | 12.8 | 24.4 | 42.1 | 59.7 | 71.4 |
|  | Female | 13.4 | 24.7 | 41.6 | 57.1 | 68.2 |
| 2nd | Male | 20.6 | 37.2 | 46.9 | 58.4 | 69.6 |
|  | Female | 21.9 | 36.5 | 45.1 | 55.9 | 66.4 |
| 3rd | Male | 21.5 | 38.1 | 44.3 | 54.1 | 65.8 |
|  | Female | 23.5 | 37.0 | 43.0 | 52.8 | 63.8 |
| 4th | Male | 21.8 | 35.1 | 41.5 | 51.3 | 63.4 |
|  | Female | 21.6 | 35.1 | 40.4 | 50.3 | 62.3 |
| 5th | Male | 19.7 | 33.6 | 39.8 | 49.8 | 62.5 |
|  | Female | 20.3 | 33.3 | 38.8 | 49.2 | 61.4 |
| 6th | Male | 19.4 | 34.4 | 40.7 | 51.4 | 64.7 |
|  | Female | 19.4 | 34.3 | 39.7 | 50.5 | 63.5 |
| 7th | Male | 19.5 | 36.4 | 42.3 | 53.1 | 66.3 |
|  | Female | 19.6 | 33.6 | 39.4 | 50.5 | 64.3 |

| Supplementary Table 5. Percentage of participants in each birth weight group with growth parameters below the 3rd percentile across the seven rounds of the infant and child health screening program | | | | | |
| --- | --- | --- | --- | --- | --- |
| Height |  |  |  |  |  |
| Screening round | Birth weight (gram) | | | | |
|  | <1,000 | 1,000–1,499 | 1,500–2,499 | 2,500–3,999 | ≥4,000 |
| 1st | 71.4 | 38.4 | 5.9 | 0.4 | 0.1 |
| 2nd | 43.1 | 16.7 | 3.5 | 0.6 | 0.1 |
| 3rd | 25.3 | 9.3 | 3.0 | 0.7 | 0.2 |
| 4th | 18.7 | 7.6 | 3.0 | 0.8 | 0.2 |
| 5th | 21.8 | 9.7 | 5.0 | 1.6 | 0.4 |
| 6th | 22.7 | 10.7 | 5.5 | 1.9 | 0.4 |
| 7th | 21.3 | 9.2 | 4.9 | 1.7 | 0.4 |
|  |  |  |  |  |  |
| Weight |  |  |  |  |  |
| Screening round | Birth weight (gram) | | | | |
|  | <1,000 | 1,000–1,499 | 1,500–2,499 | 2,500–3,999 | ≥4,000 |
| 1st | 51.1 | 22.2 | 4.3 | 0.4 | 0.1 |
| 2nd | 30.2 | 10.1 | 3.3 | 0.5 | 0.1 |
| 3rd | 28.6 | 10.1 | 4.3 | 0.9 | 0.1 |
| 4th | 32.0 | 12.6 | 6.1 | 1.4 | 0.2 |
| 5th | 36.9 | 16.6 | 8.9 | 2.4 | 0.4 |
| 6th | 35.2 | 16.6 | 8.8 | 2.6 | 0.4 |
| 7th | 31.4 | 14.3 | 7.6 | 2.3 | 0.3 |
|  |  |  |  |  |  |
| Head circumference | |  |  |  |  |
| Screening round | Birth weight (gram) | | | | |
|  | <1,000 | 1,000–1,499 | 1,500–2,499 | 2,500–3,999 | ≥4,000 |
| 1st | 48.8 | 17.6 | 2.8 | 0.3 | 0.1 |
| 2nd | 26.0 | 7.7 | 2.4 | 0.5 | 0.2 |
| 3rd | 23.8 | 7.8 | 3.4 | 0.9 | 0.3 |
| 4th | 24.6 | 8.2 | 3.9 | 1.1 | 0.3 |
| 5th | 28.4 | 9.7 | 4.7 | 1.4 | 0.4 |
| 6th | 30.5 | 10.8 | 5.7 | 1.7 | 0.5 |
| 7th | 31.9 | 10.7 | 5.7 | 1.8 | 0.4 |

| Supplementary Table 6. Percentage of participants in each birth weight group with overall developmental delays, defined as total scores <−2 SD on the Korean Developmental Screening Test for Infants and Children (K-DST), across the 2nd to 7th rounds of the infant and child health screening program | | | | | |
| --- | --- | --- | --- | --- | --- |
| Screening round | Birth weight (gram) | | | | |
|  | <1,000 | 1,000–1,499 | 1,500–2,499 | 2,500–3,999 | ≥4,000 |
| 2nd | 19.4 | 11.8 | 4.2 | 1.9 | 1.8 |
| 3rd | 16.8 | 8.0 | 3.6 | 2.1 | 2.2 |
| 4th | 16.2 | 9.1 | 4.5 | 2.9 | 3.3 |
| 5th | 13.1 | 7.1 | 3.0 | 1.8 | 2.1 |
| 6th | 14.0 | 6.5 | 2.9 | 1.7 | 1.8 |
| 7th | 15.0 | 5.4 | 2.8 | 1.7 | 1.6 |

| Supplementary Table 7. Percentage of participants in each birth weight group with developmental delays in individual domains, defined as scores < −2 SD on the Korean Developmental Screening Test for Infants and Children (K-DST), across the 2^nd^ to 7^th^ rounds of the infant and child health screening program | | | | | |
| --- | --- | --- | --- | --- | --- |
| Gross motor |  |  |  |  |  |
| Screening round | Birth weight (gram) | | | | |
|  | <1,000 | 1,000–1,499 | 1,500–2,499 | 2,500–3,999 | ≥4,000 |
| 2nd | 17.2 | 10.0 | 3.3 | 1.3 | 1.2 |
| 3rd | 13.2 | 5.7 | 1.9 | 0.8 | 0.8 |
| 4th | 12.5 | 5.7 | 2.1 | 1.0 | 1.1 |
| 5th | 11.8 | 5.8 | 2.0 | 1.0 | 1.2 |
| 6th | 13.0 | 5.6 | 2.1 | 1.0 | 1.1 |
| 7th | 14.7 | 4.9 | 2.2 | 1.2 | 1.2 |
|  |  |  |  |  |  |
| Fine motor |  |  |  |  |  |
| Screening round | Birth weight (gram) | | | | |
|  | <1,000 | 1,000–1,499 | 1,500–2,499 | 2,500–3,999 | ≥4,000 |
| 2nd | 13.6 | 7.0 | 1.9 | 0.7 | 0.6 |
| 3rd | 12.7 | 5.3 | 1.8 | 0.9 | 0.9 |
| 4th | 12.7 | 6.8 | 2.9 | 1.7 | 1.8 |
| 5th | 11.1 | 5.7 | 2.3 | 1.3 | 1.4 |
| 6th | 12.7 | 5.9 | 2.3 | 1.3 | 1.4 |
| 7th | 14.9 | 4.3 | 2.4 | 1.2 | 1.4 |
|  |  |  |  |  |  |
| Cognition |  |  |  |  |  |
| Screening round | Birth weight (gram) | | | | |
|  | <1,000 | 1,000–1,499 | 1,500–2,499 | 2,500–3,999 | ≥4,000 |
| 2nd | 13.9 | 6.9 | 1.9 | 0.7 | 0.6 |
| 3rd | 13.1 | 6.0 | 2.6 | 1.5 | 1.6 |
| 4th | 12.7 | 7.2 | 3.1 | 1.8 | 2.0 |
| 5th | 10.8 | 5.5 | 2.4 | 1.4 | 1.6 |
| 6th | 12.0 | 4.9 | 2.3 | 1.3 | 1.3 |
| 7th | 13.1 | 4.6 | 2.7 | 1.5 | 1.5 |
|  |  |  |  |  |  |
| Language |  |  |  |  |  |
| Screening round | Birth weight (gram) | | | | |
|  | <1,000 | 1,000–1,499 | 1,500–2,499 | 2,500–3,999 | ≥4,000 |
| 2nd | 15.7 | 8.3 | 2.6 | 1.1 | 1.0 |
| 3rd | 13.6 | 6.5 | 3.0 | 1.9 | 2.0 |
| 4th | 14.2 | 7.3 | 3.9 | 2.5 | 2.8 |
| 5th | 10.9 | 5.5 | 2.7 | 1.6 | 1.9 |
| 6th | 12.8 | 5.4 | 2.5 | 1.4 | 1.5 |
| 7th | 12.4 | 4.7 | 2.5 | 1.4 | 1.4 |
|  |  |  |  |  |  |
| Sociality |  |  |  |  |  |
| Screening round | Birth weight (gram) | | | | |
|  | <1,000 | 1,000–1,499 | 1,500–2,499 | 2,500–3,999 | ≥4,000 |
| 2nd | 14.7 | 7.8 | 2.5 | 1.0 | 0.9 |
| 3rd | 11.4 | 5.0 | 2.0 | 1.2 | 1.2 |
| 4th | 12.9 | 6.7 | 3.1 | 1.9 | 2.1 |
| 5th | 10.4 | 5.4 | 2.4 | 1.4 | 1.7 |
| 6th | 10.6 | 4.5 | 2.0 | 1.2 | 1.3 |
| 7th | 10.9 | 3.9 | 2.2 | 1.2 | 1.3 |
|  |  |  |  |  |  |
| Self-care |  |  |  |  |  |
| Screening round | Birth weight (gram) | | | | |
|  | <1,000 | 1,000–1,499 | 1,500–2,499 | 2,500–3,999 | ≥4,000 |
| 3rd | 11.5 | 5.3 | 2.1 | 1.1 | 1.1 |
| 4th | 13.3 | 6.8 | 2.9 | 1.7 | 1.9 |
| 5th | 10.9 | 5.5 | 2.2 | 1.2 | 1.4 |
| 6th | 11.3 | 4.8 | 1.9 | 1.1 | 1.1 |
| 7th | 12.9 | 4.0 | 2.3 | 1.2 | 1.2 |

| Supplementary Table 8. Number of participants with complete growth parameter records by screening round, sex, and birth weight group | | | | | | | |
| --- | --- | --- | --- | --- | --- | --- | --- |
| Screening round | Sex | Birth weight (gram) | | | | | Total (n) |
|  |  | <1,000 | 1,000–1,499 | 1,500–2,499 | 2,500–3,999 | ≥4,000 |  |
| 1st | Males | 550 | 2,766 | 52,052 | 1,075,360 | 55,228 | 1,185,956 |
|  | Females | 649 | 2,764 | 28,313 | 1,017,094 | 64,101 | 1,112,921 |
|  | Total | 1,199 | 5,530 | 80,365 | 2,092,454 | 119,329 | 2,302,877 |
| 2nd | Males | 802 | 3,158 | 48,390 | 998,565 | 52,496 | 1,103,411 |
|  | Females | 931 | 3,089 | 26,359 | 949,522 | 61,046 | 1,040,947 |
|  | Total | 1,733 | 6,247 | 74,749 | 1,948,087 | 113,542 | 2,144,358 |
| 3rd | Males | 1,093 | 3,508 | 44,388 | 957,341 | 48,173 | 1,054,503 |
|  | Females | 1,253 | 3,426 | 24,339 | 914,530 | 55,711 | 999,259 |
|  | Total | 2,346 | 6,934 | 68,727 | 1,871,871 | 103,884 | 2,053,762 |
| 4th | Males | 1,176 | 3,094 | 36,735 | 800,757 | 39,857 | 881,619 |
|  | Females | 1,301 | 3,022 | 20,274 | 768,424 | 46,051 | 839,072 |
|  | Total | 2,477 | 6,116 | 57,009 | 1,568,181 | 85,908 | 1,719,691 |
| 5th | Males | 936 | 2,540 | 28,881 | 631,576 | 31,256 | 695,189 |
|  | Females | 1,097 | 2,448 | 15,945 | 607,947 | 36,049 | 663,486 |
|  | Total | 2,033 | 4,988 | 44,826 | 1,239,523 | 67,305 | 1,358,675 |
| 6th | Males | 680 | 1,751 | 19,956 | 441,991 | 21,637 | 486,015 |
|  | Females | 793 | 1,703 | 10,988 | 425,095 | 24,999 | 463,578 |
|  | Total | 1,473 | 3,454 | 30,944 | 867,086 | 46,639 | 949,596 |
| 7th | Males | 445 | 1,092 | 12,752 | 288,221 | 13,844 | 316,354 |
|  | Females | 507 | 1,052 | 6,948 | 276,827 | 15,988 | 301,322 |
|  | Total | 952 | 2,144 | 19,700 | 565,048 | 29,832 | 617,676 |

| Supplementary Table 9. Number of participants with available K-DST results by screening round and birth weight group | | | | | | |
| --- | --- | --- | --- | --- | --- | --- |
| Screening round | Birth weight (gram) | | | | | Total (n) |
|  | <1,000 | 1,000–1,499 | 1,500–2,499 | 2,500–3,999 | ≥4,000 |  |
| 2nd | 1,528 | 5,492 | 97,869 | 1,651,806 | 63,160 | 1,819,855 |
| 3rd | 2,279 | 6,797 | 101,198 | 1,812,109 | 66,539 | 1,988,922 |
| 4th | 2,476 | 6,116 | 85,904 | 1,568,107 | 57,004 | 1,719,607 |
| 5th | 2,033 | 4,987 | 67,302 | 1,239,429 | 44,822 | 1,358,573 |
| 6th | 1,473 | 3,454 | 46,632 | 866,998 | 30,941 | 949,498 |
| 7th | 951 | 2,143 | 29,823 | 564,883 | 19,697 | 617,497 |
